# Neurocognitive moderation of repetitive transcranial magnetic stimulation (rTMS) effects on cannabis use in schizophrenia: a preliminary analysis

**DOI:** 10.1038/s41537-022-00303-2

**Published:** 2022-11-17

**Authors:** Samantha Johnstone, Darby J. E. Lowe, Karolina Kozak-Bidzinski, Marcos Sanches, David J. Castle, Jennifer S. Rabin, Rachel A. Rabin, Tony P. George

**Affiliations:** 1 Centre for Complex Interventions and Addictions Division, Centre for Addiction and Mental Health, Toronto, ON M6G 1H4 Canada; 2 Temerty Faculty of Medicine, University of Toronto, Toronto, ON Canada; 3 Division of Neurology, Department of Medicine, Sunnybrook Health Sciences Centre, University of Toronto, Toronto, ON M4N 3M5 Canada; 4 Harquail Centre for Neuromodulation, Hurvitz Brain Sciences Program, Sunnybrook Research Institute, Toronto, ON M4N 3M5 Canada; 5 Rehabilitation Sciences Institute, University of Toronto, Toronto, M5G 1V7 Canada; 6 Department of Psychiatry, McGill University, Montreal, QC Canada

**Keywords:** Schizophrenia, Human behaviour

## Abstract

Repetitive transcranial magnetic stimulation (rTMS) is a promising treatment for cannabis use disorder in schizophrenia; however, gaps in the literature remain as to the potential role of neurocognitive functioning in treatment response. We evaluated the moderating role of select cognitive functions including baseline executive functioning, verbal memory, and sustained attention, and we explore the mediating role of changes in task performance on changes in cannabis use in both active and sham rTMS groups. Participants underwent high-frequency (20 Hz) rTMS applied to the bilateral dorsolateral prefrontal cortex 5x/week for 4 weeks. Weekly self-report of cannabis use and semi-quantitative urinary carboxy-tetrahydrocannabinol levels were recorded. A neurocognitive battery assessing verbal memory, visuospatial working memory, verbal working memory, sustained attention, delayed discounting, and complex planning was administered pre- and post-treatment. Better baseline performance on tasks assessing sustained attention, delayed discounting, and complex planning moderated the extent to which participants in the active group reduced cannabis use. There were no significant indirect pathways between treatment, changes in neuropsychological performance, and changes in cannabis use; however, active rTMS improved complex planning and sustained attention. These preliminary findings suggest that there is a moderating role of sustained attention, delayed discounting, and complex planning on the effects of rTMS on cannabis use. Further, mediation models suggest rTMS may exert direct effects on cannabis use independent of its effects on cognitive functioning in people with SCZ. Trial Registration: clinicaltrials.gov: NCT03189810.

## Introduction

Schizophrenia (SCZ) is a chronic psychotic disorder associated with significant impairment in a wide range of cognitive functions, including executive function. The latter is manifested clinically by deficits in attaining goals, solving complex problems, memory and attention^1^. SCZ is highly comorbid with cannabis use disorder (CUD) with a prevalence of ~26%^2^, and linked to poorer clinical and cognitive outcomes^3^. Executive dysfunction in SCZ impairs patient engagement with psychological and pharmacological treatment for SCZ^4,5^, thus potentially reducing efficacy of typical CUD treatments and impairing recovery attempts.

Amongst people with substance use disorders, cognitive functioning predicts several indicators of successful addiction recovery. For example, better performance on executive function and verbal memory tasks (which is strongly related to executive functioning capacity^6^) predict greater motivation for changes in substance use behavior^7^, whereas poorer performance predicts greater denial in the need for substance use change^8^. Similarly, sustained attention predicts motivation for substance use behavior change^9^. In addition, intact executive function has been correlated with recovery-based self-efficacy and participation in therapy for substance use^10,11^. Furthermore, poorer delayed discounting and inability to delay gratification or control impulses were related to reduced capacity to learn effective coping skills for substance use^12^, whereas deficits in working memory predict substance use relapse after treatment^13^. As such, preserved cognitive functioning, particularly in aspects of executive function may play a role in addiction treatment response^14^.

There is extensive overlapping neurobiology of SCZ and CUD, including dysregulated gamma aminobutyric acid activity (GABA), and catechol-O-methyltransferase (COMT) allelic gene variation, and dysfunctional endocannabinoid system, particularly a greater density of cannabinoid receptor type 1 (CB1Rs) in the dorsolateral prefrontal cortex (DLPFC^15^), which contributes to dysregulated reward system functioning in addiction^16^. Repetitive transcranial magnetic stimulation (rTMS) represents a promising treatment that may target CUD while bypassing cognition-related difficulties in treatment engagement for SCZ patients^17^ and has dual potential as an intervention for both disorders^17–19^. In comorbid CUD and SCZ, rTMS applied to the DLPFC may regulate subcortical activity (e.g., glutamateric, GABAergic, and dopaminergic neurotransmission in the mesolimbic pathway), as is seen in addiction without SCZ^20,21^. Regulation of subcortical activity may directly reduce cravings for cannabis as well as improve top-down cognitive control processes, and thus inhibition of craving and consumption of substances^17^.

In a case-series with non-psychiatric participants with CUD, rTMS showed efficacy in reducing cravings for cannabis and amount of cannabis used weekly^22^, although evidence with respect to changes in cannabis cravings are mixed^23^. Furthermore, in individuals with SCZ, our preliminary randomized controlled trial found trending treatment x time effects after 4 weeks of active versus sham treatment on cannabis use outcomes in people with SCZ^19^. Specifically, there was a medium to large effect size in between-group differences in reduction of cannabis grams per day (GPD; *d* = 0.72) and a medium effect of tetrahydrocannabinol (THC) content in urine (*d* = 0.55) in the active group relative to the sham group^19^. Furthermore, significant treatment x time interactions were found for improvements in the active group in cannabis craving, positive and general SCZ symptoms, depressive symptoms, and certain domains of cognition (e.g., sustained attention). Thus, rTMS may be a promising intervention for CUD in SCZ.

Due to gaps in the current literature, there are several questions that remain unanswered. (1) Are rTMS effects on reductions in cannabis use in SCZ dependent on better baseline cognitive functioning as is seen in behavioral and pharmacological treatment, and (2) Does rTMS improve neuropsychological performance and does this partially explain reductions in cannabis use? We conducted a secondary analysis of data from the rTMS treatment study in comorbid SCZ and CUD described above to assess the hypothesis that better baseline executive function, and in particular, delayed discounting and complex planning skills, results in greater cannabis reductions in the active rTMS group. Further, an exploratory analysis was conducted to ascertain hypothesized models of change. Specifically, we assess whether active rTMS induces changes in select cognitive functions that are hypothesized to be responsive to non-invasive neuromodulation, mediate the relationship between rTMS treatment and changes in cannabis use; namely, verbal memory, visuospatial working memory, verbal working memory, planning, and sustained attention^17^.

## Results

### Effect of baseline neuropsychological performance on cannabis outcomes

#### Delayed discounting

We found a significant interaction between treatment, time, and geometric K scores (*d* = −0.62, [−1.19, −0.04]), such that in the active group, smaller geometric K scores (i.e., better performance) predicted lower THC urinalysis content at the end of treatment (see Table 1), whereas in the sham group, smaller geometric K scores predicted greater THC urinalysis content at end of treatment. There was no three-way interaction with geometric K scores on GPD.

**Table 1:** Results of linear mixed-effects models fit with treatment, time, and baseline neuropsychological performance on THC content in urine.

Neuropsychological measure	Fixed effects	*Estimate*	*SE*	*df*	*t*	*p*
*NarcoCheck (ng/mL)*						
HVLT-R composite	Intercept	343.89	50.13	18.13	6.86	<0.001***
	Treatment	−14.72	50.13	18.13	−0.29	0.77
	**Time**	**54.03**	**21.03**	**16.70**	**2.57**	0.020*
	HVLT-R	−33.40	51.60	35.82	−0.65	0.52
	Treatment x Time	31.40	21.03	16.70	1.49	0.15
	Treatment x HVLT-R	−88.43	51.60	35.82	−1.71	0.095
	Time x HVLT-R	−24.01	30.56	18.24	−0.79	0.44
	Treatment x Time x HVLT-R	−28.54	30.56	18.24	−0.93	0.36
SDR composite	Intercept	348.11	48.98	18.80	7.11	<0.001***
	Treatment	−28.83	48.98	18.80	0.59	0.56
	**Time**	**51.51**	**22.61**	**17.82**	**2.28**	0.035*
	SDR	12.81	55.77	30.18	0.23	0.82
	Treatment x Time	38.52	22.61	17.82	1.70	0.11
	Treatment x SDR	−18.63	55.77	30.18	−0.33	0.74
	Time x SDR	41.02	39.40	18.21	1.04	0.31
	Treatment x Time x SDR	63.47	39.40	18.21	1.61	0.12
CPT attentional index	Intercept	375.15	40.05	14.94	9.37	<0.001***
	Treatment	2.39	40.05	14.94	0.06	0.95
	Time	49.15	23.42	13.18	2.10	0.055
	CPT	42.21	21.93	23.50	1.92	0.066
	Treatment x Time	34.11	23.42	13.18	1.46	0.16
	**Treatment x CPT**	**−69.87**	**21.93**	**23.50**	**−3.19**	<0.01**
	Time x CPT	−5.98	15.12	16.02	−0.40	0.69
	Treatment x Time x CPT	−5.21	15.12	16.02	−0.34	0.73
CPT hit rate	Intercept	604.68	363.62	32.85	1.66	0.11
	Treatment	106.31	363.62	32.85	0.29	0.77
	Time	64.71	193.93	18.04	0.33	0.74
	CPT	−0.59	0.90	32.71	−0.65	0.52
	Treatment x Time	94.30	193.93	18.04	0.49	0.63
	Treatment x CPT	−0.25	0.91	32.71	−0.27	0.79
	Time x CPT	−0.04	0.49	18.73	−0.09	0.93
	Treatment x Time x CPT	−0.18	0.49	18.73	−0.37	0.72
DS forward	Intercept	892.16	126.93	31.05	7.03	< 0.001***
	Treatment	45.56	126.93	31.05	0.36	0.72
	Time	−10.40	61.20	14.70	−0.17	0.87
	**DS**	**−52.31**	**10.90**	**22.76**	**−4.80**	<0.001***
	**Treatment x Time**	**196.69**	**61.20**	**14.70**	**3.21**	0.005**
	Treatment x DS	−5.04	10.90	22.76	−0.46	0.65
	Time x DS	4.67	5.76	14.61	0.81	0.43
	**Treatment x Time x DS**	**−17.07**	**5.76**	**14.61**	**−2.96**	0.01**
TOL total correct	Intercept	663.55	347.73	11.71	1.91	0.08
	Treatment	−185.48	347.73	11.71	−0.53	0.60
	Time	450.77	218.34	8.63	2.07	0.07
	TOL	−5.0	3.62	10.96	−1.38	0.20
	**Treatment x Time**	**−601.89**	**218.34**	**8.63**	**−2.76**	0.023*
	Treatment x TOL	2.85	3.62	10.96	0.79	0.45
	Time x TOL	−4.98	2.34	8.85	−2.13	0.062
	**Treatment x Time x TOL**	**6.49**	**2.34**	**8.85**	**2.77**	0.022*
KDDT Geometrick K	Intercept	256.04	101.30	25.94	2.53	0.02*
	**Treatment**	**−297.53**	**101.30**	**25.94**	**−2.94**	0.007**
	Time	−13.33	72.86	13.85	−0.18	0.85
	Geo K	−15.64	21.23	14.54	−0.74	0.47
	Treatment x Time	−134.64	72.85	13.85	−1.85	0.08
	**Treatment x Geo K**	**−80.39**	**21.23**	**14.54**	**−3.79**	0.001**
	Time x Geo K	−9.54	18.29	13.83	−0.52	0.61
	**Treatment x Time x Geo K**	**−42.20**	**18.29**	**13.83**	**−2.31**	0.037*

Hopkins Verbal Learning Test (HVLT-R), the Spatial Delayed Response (SDR), Continuous Performance Test (CPT), Digit Span (DS), Tower of London (TOL), Kirby Delay-Discounting Task (KDDT).

Bold values in the table represent significant interactions of interest (*p* < 0.05)

*p < 0.05; **p < 0.01; ***p < 0.001

#### Complex planning

We observed a significant three-way interaction between treatment, time, and better accuracy performance on the TOL, such that in the active group, greater TOL total correct scores predicted reductions in GPD (*d* = −1.86, [0.71, 2.97]) and THC content in urinalysis (*d* = −0.93, [0.13, 1.70]) at the end of treatment, whereas this was not the case in the sham group (see Table 2). Conversely, longer problem-solving time on the TOL was associated with greater reductions in GPD (*d* = 1.40, [0.37, 2.39]) as was requiring more moves to complete the puzzle (*d* = 1.04, [0.21, 1.84]), constituting lower efficiency. Notably, participants with longer problem solving times had more correct towers (*r* = 0.93). There were no interaction effects of TOL initiation on GPD or THC content in urine.

**Table 2:** Results of linear mixed-effects models fit with treatment, time, and baseline neuropsychological performance on cannabis grams per day.

Neuropsychological measure	Fixed effects	*Estimate*	*SE*	*df*	*t*	*p*
*Grams per day*						
HVLT-R composite	Intercept	0.60	0.09	18.08	6.61	0.000***
	Treatment	−0.97	0.09	18.08	−1.06	0.30
	**Time**	**0.18**	**0.05**	**17.06**	**3.43**	0.003**
	HVLT-R	−0.02	0.11	33.10	−0.15	0.88
	Treatment x Time	0.07	0.05	17.06	1.33	0.20
	Treatment x HVLT-R	−0.17	0.11	33.10	−1.55	0.13
	Time x HVLT-R	−0.03	0.08	19.61	−0.49	0.63
	Treatment x Time x HVLT-R	−0.08	0.08	19.61	−1.00	0.32
SDR composite	Intercept	0.59	0.11	17.10	5.57	<0.001***
	Treatment	−0.10	0.11	17.10	−0.92	0.37
	**Time**	**0.17**	**0.05**	**17.70**	**3.70**	<0.001***
	SDR	<0.01	0.12	29.05	−0.07	0.92
	**Treatment x Time**	**0.10**	**0.04**	**17.70**	**2.15**	0.045*
	Treatment x SDR	−0.07	0.12	29.05	−0.68	0.50
	**Time x SDR**	**0.23**	**0.08**	**18.40**	**2.84**	0.01*
	Treatment x Time x SDR	−0.16	0.08	18.40	−1.97	0.06
CPT hit rate	Intercept	1.22	0.75	29.73	1.63	0.11
	Treatment	−0.82	0.75	29.73	−1.10	0.28
	Time	−0.62	0.46	20.03	−1.32	0.19
	CPT	<−0.01	<0.01	29.52	−0.81	0.42
	Treatment x Time	0.13	0.46	20.03	0.27	0.78
	Treatment x CPT	<0.01	<0.01	29.52	0.98	0.33
	Time x CPT	<0.01	<0.01	20.54	1.66	0.10
	Treatment x Time x CPT	<0.01	<0.01	20.54	−0.18	0.85
CPT attentional index	Intercept	0.64	0.08	19.74	7.84	<0.001***
	Treatment	−0.09	0.08	19.74	−1.16	0.81.26
	**Time**	**0.18**	**0.06**	**18.43**	**3.01**	0.007**
	**CPT**	**0.12**	**0.05**	**27.15**	**2.62**	0.01*
	Treatment x Time	0.07	0.06	18.43	1.26	0.22
	**Treatment x CPT**	−**0.13**	**0.05**	**27.15**	−**2.82**	0.009**
	Time x CPT	0.01	0.04	22.52	0.15	0.88
	Treatment x Time x CPT	−0.02	0.04	22.52	−0.49	0.61
TOL total correct	Intercept	−0.03	0.16	14.72	−0.19	0.029*
	**Treatment**	**−0.64**	**0.16**	**14.72**	**−4.12**	<0.001***
	**Time**	**0.35**	**0.07**	**8.0**	**4.52**	0.05*
	**TOL**	**<0.001**	<**0.001**	**8.19**	**2.87**	0.01*
	**Treatment x Time**	**−0.30**	**0.07**	**8.02**	**−3.84**	<0.001***
	**Treatment x TOL**	**<−0.01**	**<0.001**	**8.19**	**5.75**	<0.001***
	**Time x TOL**	**<−0.01**	**<0.01**	**8.05**	**2.31**	<0.05*
	**Treatment x Time x TOL**	<**0.01**	**<0.001**	**8.05**	**5.29**	<0.001***
TOL total moves	Intercept	−0.13	0.35	11.80	−0.36	0.72
	Treatment	0.26	0.35	11.80	−0.75	0.47
	Time	0.01	0.17	7.29	0.08	0.94
	TOL	<0.01	<0.01	10.85	1.43	0.18
	**Treatment x Time**	**−0.51**	**0.17**	**7.29**	**−3.02**	0.01**
	Treatment x TOL	<0.01	<0.01	10.85	1.08	0.30
	Time x TOL	<0.01	<0.01	7.32	0.96	0.36
	**Treatment x Time x TOL**	<0.01	<0.01	**7.32**	**3.79**	<0.006**
TOL problem solving time	Intercept	−1.12	0.75	15.96	1.49	0.15
	Treatment	−0.23	0.75	15.96	−0.30	0.77
	Time	−0.45	0.49	9.03	−0.92	0.38
	TOL	0.02	0.08	15.96	1.97	0.06
	**Treatment x Time**	**−1.44**	**0.49**	**9.03**	**−2.91**	0.017*
	Treatment x TOL	<0.01	0.08	15.96	0.40	0.69
	Time x TOL	<0.01	<0.01	8.80	1.26	0.24
	**Treatment x Time x TOL**	**0.02**	<0.01	**8.80**	**3.09**	0.013*
KDDT Geometric K	Intercept	0.81	0.36	25.78	2.25	0.03*
	Treatment	−0.29	0.36	25.78	−0.83	0.41
	Time	0.26	0.33	18.75	0.80	0.44
	KDDT	0.05	0.09	24.14	0.60	0.55
	Treatment x Time	0.18	0.33	18.75	0.56	0.58
	Treatment x KDDT	−0.06	0.09	24.14	−0.62	0.54
	Time x KDDT	0.02	0.08	18.78	0.27	0.79
	Treatment x Time x KDDT	0.03	0.08	18.78	0.32	0.75

Hopkins Verbal Learning Test (HVLT-R), the Spatial Delayed Response (SDR), Continuous Performance Test (CPT), Tower of London (TOL), Kirby Delayed Discounting Task (KDDT).

Bold values in the table represent significant interactions of interest (*p* < 0.05)

*p < 0.05; **p < 0.01; ***p < 0.001

#### Visuospatial working memory

We observed a trending interaction between treatment, time, and SDR composite scores (*d* = −0.46, [−0.93, 0.03]), such that in the active group, worse baseline SDR performance was associated with greater reductions in GPD. There were no significant interactions between treatment, time, and SDR scores on THC content in urine.

#### Verbal working memory

We found a significant interaction between treatment, time, and baseline performance on the Digit Span Forward task (*d* = −0.77, [−1.35, −0.18]), such that worse performance on the Digit Span Forward predicted greater reductions on THC content in urine from baseline to end of treatment in the active group. There were no interaction effects between treatment, time, and baseline performance on the Digit Span Backward task on THC content in urine nor the Digit Span task with time or treatment on GPD (Supplementary Table).

#### Sustained attention

Across both groups there was a trending moderation effect of better performance on CPT hit rate scores on reductions in GPD from baseline to end of treatment (time x CPT *d* = −0.46, [−0.93, 0.03]). Post hoc analyses showed higher CPT hit rate scores were related to greater reductions in GPD treatment in the active group (*d* = 0.36) as well as the sham group (*d* = 0.39). While there was no treatment x time interactions with CPT attentional index scores, we did observe a significant interaction between treatment and CPT attentional scores on GPD (*d* = −054, [−0.94, −0.13]) and THC content in urine (*d* = −0.66, [−1.10, −0.21]). Based on observation of the data (Supplementary Figure), reductions in cannabis use in the active group did not differ as a function of CPT attention index scores. There were no treatment x time interactions with CPT variability scores (Supplementary Table).

#### Memory

There were no significant interactions between treatment, time, and effects of baseline HVLT-R composite scores on cannabis GPD (*d* = −0.23, [−0.67, 0.23]) nor on THC content in urine (*d* = −0.22, [−0.68, 0.25]).

### Models of change analysis

**Fig. 1:**
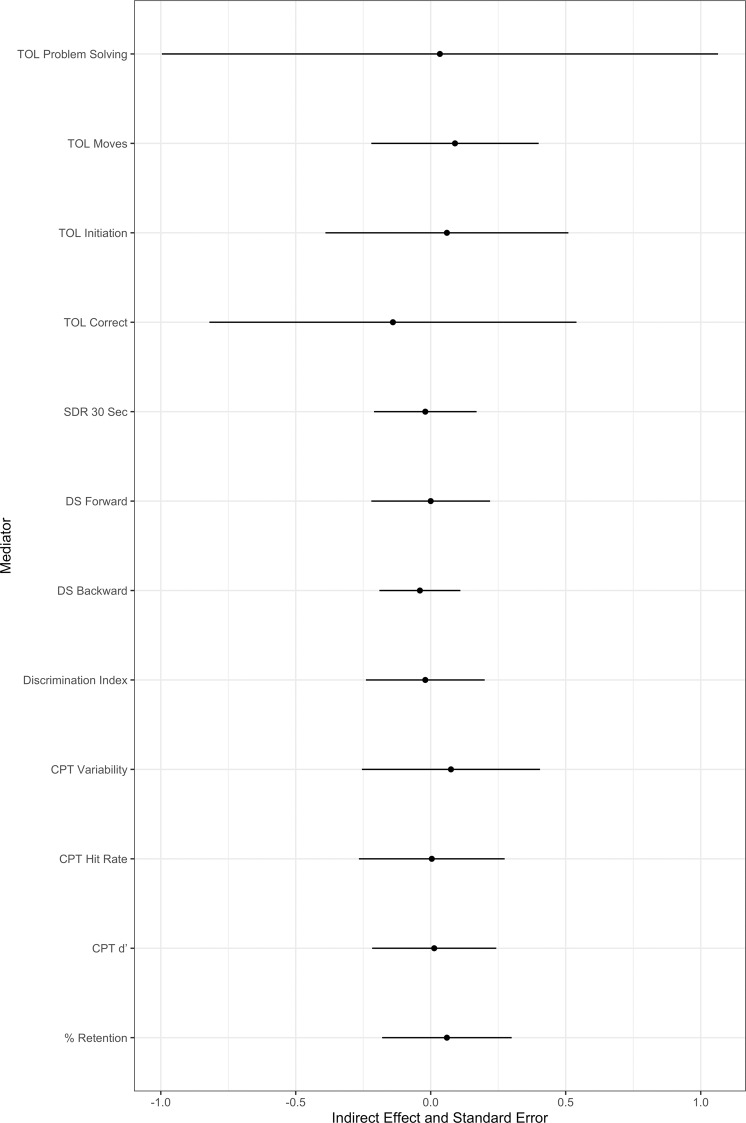
Models of change neurocognitive mediation plot. Models of change figure depicting standardized effect sizesalong with standard error of the mean estimates for indirect pathways through select neurocognitive functions as amediator between rTMS treatment and changes in cannabis use.

To explore predicted models of change, we analyzed the mean change for the tasks hypothesized to change from baseline to Day 28 (see Fig. 1), for both the active and sham treatment groups as a mediator between treatment group and reductions in GPD. In the models of change, there was a small to medium effect of treatment group on improvements in HVLT-R percent retention (*a* = 0.29, [−0.23, 0.82]), discrimination index (*a* = 0.27, [−0.79, 0.26]), CPT attentional index (*a* = 0.25, [−0.30, 0.80]), and a medium effect in improvements in TOL time to initiation (*a* = −0.44, [−1.29, 0.40]), CPT hit rate (*a* = 0.48, [−0.01, 0.98]) CPT variability (*a* = −0.58, [−1.04, −0.12]), and TOL problem solving (*a* = 0.47, [−0.37, 1.31]). Moreover, there was a small effect of improvements in DS backward (*b* = 0.20, [−0.35, 0.75]), DS Forward (*b* = 0.24, [−0.28, 0.78]), and a medium effect of improvements in TOL total moves (*b* = −0.30, [−0.71, 0.11]) on changes in cannabis GPD. There was also a medium effect of TOL total correct (*b* = −0.42, [−0.65, −0.19]), but not in the predicted direction. The full indirect pathway (AB) analyses were not significant in any of the above models; Fig. 1 displays effect sizes of indirect pathways and standard error estimates. Finally, to ascertain directionality of predicted models of change, mediator and outcome variables were swapped, such that we assess whether changes in cannabis use predicts changes in neurocognitive functioning. Full indirect pathways (AB) were not significant in any of the models; however, changes in cannabis use had a medium to large effect on changes in TOL total moves (*ab* = 0.51, [−0.26, 1.96], *p* = 0.19) and total correct (*ab* = 0.72, [0.11, 1.63], *p* = 0.07).

## Discussion

The literature suggests overlapping neuropathophysiology between CUD and SCZ, as well as intersecting neurobiology associated with deficits in executive functioning experienced in both disorders (i.e., dysfunctional DLPFC^24,25^). Despite the high prevalence of CUD in SCZ, there remains a lack of consensus on potential treatments for this comorbidity. Thus, we investigated the role of neurocognitive performance on rTMS-induced changes in cannabis use. As expected, better baseline complex planning and delayed discounting abilities in SCZ predicted greater rTMS-induced changes in cannabis use over time, as measured by self-reported cannabis use and urinary THC-COOH. However, changes in neurocognitive performance did not mediate the efficacy of rTMS on changes in cannabis use. It is possible that substantial improvements in cognitive performance as a result of rTMS may require longer treatment periods to have a significant influence on cannabis use^17^.

Taken together, higher response inhibition (CPT hit rate), complex planning accuracy, and delayed discounting all predicted reduced cannabis use in the active rTMS group. Although complex planning efficiency was negatively correlated to reduced GPD in the active group (e.g., participants who took longer to plan moves experienced greater improvements), other research has found that in healthy samples, longer planning times predicted a greater number of “perfect solutions”^26^ and as such may not reflect a deficit in complex planning. Similarly, participants in this sample who took longer to solve the problem were also more likely to have greater accuracy on the TOL. Significant moderation effects observed in the active rTMS group suggest select baseline cognitive abilities might have influenced the extent to which rTMS reduces cannabis use, either directly or indirectly through encouraging engagement in behavioral treatment sessions. Poorer delayed discounting, response inhibition, and complex planning skills may reflect a more severe SCZ profile that influences the degree to which rTMS is effective through how much an individual is able or willing to participate in recovery, inhibit impulses to use drugs, and maintain long-term goals. Thus, integrating supportive therapies that help individuals overcome cognitive deficits (e.g., cognitive remediation strategies)^27^ may facilitate the effects of neuromodulation on CUD outcomes in SCZ.

Notably, in the sham group some participants reduced cannabis use with the help of weekly behavioral support sessions; our moderation analyses suggest that this primarily relied on better select baseline cognitive functions. Conversely in the active group, improvements were seen regardless, albeit to varying extents dependent on delayed discounting, response inhibition, and complex planning skills (Supplementary Figure). This indicates rTMS may act independently of some aspects of cognitive performance to reduce cannabis use in SCZ. Executive dysfunction in SCZ traditionally represents a significant hindrance to complex behaviors such as addiction recovery as they are associated with greater risk-taking, more impulsivity, difficulty setting and maintaining goals, and poor cognitive flexibility^1,28^.

Delayed discounting, complex planning, and response inhibition may have a moderating effect on rTMS effects that is not observed from other cognitive tasks, due to their close relation to substance use behaviors (e.g., making choices now to abstain for greater future goals like recovery, over choosing immediate reward like the high from the substance, “urge surfing” cravings), as well as creating and following recovery plans^12^. Furthermore, although studies have found better baseline verbal learning and memory, working memory, and attention to predict greater success in recovery^14^, these skills may have been more amenable to rTMS and thus baseline performance was not predictive of changes in cannabis use. Indeed, our mediation models found medium treatment effects on the HVLT-R and CPT attentional index, potentially indicating treatment-associated compensation for these skills in reducing cannabis use. It is also possible that these tests were less sensitive in assessing target domains. However, we recognize that this study has a small sample size and hope to replicate these findings in a larger sample. Nonetheless, we believe this is relevant to the scientific community for directing future studies.

While the indirect effect of neuropsychological changes predicting changes in cannabis use was not significant, we were underpowered for mediation analysis, and it was an exploratory undertaking to see potential paths that explain individual differences in cannabis use outcomes after rTMS treatment related to neuropsychological underpinnings of SCZ. Future research should assess the extent to which neuropsychological changes influence rTMS effects on cannabis use, given findings that baseline performance significantly affects the degree to which rTMS is effective in reducing cannabis use.

Nonetheless, there were several notable findings pertinent to the model of change hypothesis. There was a medium effect of active treatment on improvements in sustained attention, complex planning, as well as verbal learning and memory, supporting previous research showing cognitive improvements in SCZ after rTMS treatment^17^. In SCZ, reduced cortical activity results in a lack of cortical inhibitory control, impairing suppression of hyperactive automatic tendencies that maintain addiction^1,14,29^. Given dysfunctional frontal-subcortical circuitry in SCZ, co-occurring CUD worsens cognitive impairment, further hindering recovery attempts^30–34^. That is, overstimulation of widespread dysregulated CB1Rs leads to aberrant neurotransmission, such as blunted dopaminergic activity in subcortical regions and cognitive decline, making it difficult to sustain long-term goals like abstinence^35,36^. Encouragingly, the application of rTMS to the DLPFC facilitates the induction of temporary electric currents and normalizes cortical excitability in this region^37^. Normalization of activity in the DLPFC may lead to enhanced cognitive control, reducing cannabis use behavior. Furthermore, changes in sustained attention and complex planning had a medium effect on reductions in cannabis use. These skills may facilitate engagement with supportive behavioral interventions delivered alongside rTMS^4,5^, potentially amplifying benefits of behavioral interventions. Secondary outcomes of normalized activity in reward processing centers and pathways may further reduce cannabis use in combination with influences from behavioral support. Indeed, we also found small to medium effects of changes of verbal working memory, and complex planning on changes in cannabis use. Hypothesized improvements in cognitive functioning related to rTMS in SCZ and relations to engagement in behavioral interventions may be established by comparing rTMS responses with and without the behavioural counselling intervention.

### Limitations

The main limitation of this study is the small sample size. Indeed, the study was underpowered and results should be considered preliminary; results should be replicated in a larger sample size for generalizability of findings. Individuals with SCZ exhibit reduced motivation in quitting or reducing cannabis use and experience paranoia about new treatments as well as accessibility issues with attending in-person sessions, such that a multi-site trial may enhance recruitment efforts. Moreover, we did not correct for multiple comparisons for the various neuropsychological analyses conducted as our analyses were exploratory. Notably, previous studies in people with schizophrenia have found no cannabis-abstinence induced improvements in Digit Span, CPT, SDR, or the KDDT, suggesting any improvements in this sample to be a direct result of rTMS. However, it is still possible that changes in rTMS-induced changes in cannabis use may have resulted in changes in select cognitive functions (e.g., HVLT^38^) or the TOL. The direction of change is not conclusive without a larger sample or comparison of neurocognitive functions that reduce cannabis use in response to active (versus sham) rTMS treatment, relative to those who do not. Furthermore, it is possible practice effects played a role in changes in neurocognitive performance from baseline to end of treatment; however, please note that a training session that was not analyzed was used prior to baseline to reduce the presence of practice effects. In addition, use of brain-based measurements (e.g., positron emission topography, magnetic resonance imaging, electroencephalogram) may be informative to understanding neurobiological changes occurring as a result of rTMS and whether these are associated with changes in neurocognitive functions and cannabis use. Finally, there was a significant effect of time in both groups, potentially indicative of regression to the mean, or a result of cannabis behavioral sessions received by both groups. Nonetheless, differences between treatment groups support efficacy of rTMS for treating CUD in individuals with SCZ as well as the relevance of neurocognitive functioning to treatment outcomes.

## Conclusions

We found significant moderating effects of baseline delayed discounting, response inhibition, and complex planning that may explain the effects of high-frequency rTMS on cannabis use outcomes in SCZ. However, mediation of cannabis use outcomes by rTMS-related improvements in cognition was not significant. Our findings support the importance of cognitive deficits in understanding rTMS effects on cannabis use in SCZ. Further research with larger samples is needed to determine whether rTMS-related changes in cognitive functioning in SCZ may influence cannabis use outcomes in a clinically significant manner.

## Methods

### Participants

We recruited 19 individuals (18–55 years) diagnosed with SCZ or schizoaffective disorder and co-occurring CUD with the Structured Clinical Interview for the DSM-5; CUD severity was ascertained using the Cannabis Use Disorder Identification Test-Revised (CUDIT-R). We required participants to have a positive urine drug test for THC-COOH on MEDTOX®, and NarcoCheck® semi-quantitative levels ≥150 ng/ml at baseline to indicate current cannabis use (see ref. ^19^). Exclusion criteria were other substance use (other than caffeine, non-disordered alcohol use, or nicotine), suicidal ideation, a history of seizures, or previous rTMS treatment (CONSORT diagram can be found in ref. ^19^). Sample demographics and clinical characteristics are reported in Table 3. Cannabis Timeline Follow-back and NarcoCheck® were administered weekly to assess changes in self-reported cannabis GPD and in semi-quantitative THC-COOH content present in urine, respectively^19^. All participants gave written informed consent, as per the Centre for Addiction and Mental Health Research Ethics Board.

**Table 3:** Sample baseline demographic and clinical characteristics.

Measure	Group		*p*
	Active rTMS (*n* = 9)	Sham (*n* = 10)	
Diagnosis (SCZ/SA)	7/2	7/3	ns
Age of SCZ diagnosis (years)	23.89 ± 4.70	20.40 ± 2.07	0.05
Atypical/typical/both AP	6/0/3	8/2/0	ns
Age (years)	34.78 ± 10.35	29.10 ± 5.38	0.15
Gender (M/F)	9/0	9/1	ns
Ethnicity (White/Black/Hispanic/Other/Mixed)	5/3/1/0/0	4/3/0/1/2	ns
Education (years)	12.56 ± 2.60	12.35 ± 1.63	0.24
WTAR IQ Score	105.89 ± 9.25	105.20 ± 5.96	0.85
PANSS positive	13.78 ± 3.42	12.20 ± 3.58	0.34
PANSS negative	14.67 ± 4.61	13.60 ± 3.60	0.58
PANSS general	26.00 ± 5.70	24.10 ± 5.02	0.45
PANSS total	54.44 ± 10.01	49.90 ± 8.25	0.29
CUD severity (moderate/severe)	4/5	4/6	ns
Grams per day	0.64 ± 0.27	1.32 ± 1.94	0.31
NarcoCheck® THC-COOH (ng/mL)	385.53 ± 222.62	360.00 ± 214.48	0.80
CUDIT-R (*n* = 6,6)	16.17 ± 6.97	17.83 ± 5.12	0.65
Joint-Years	10.37 ± 7.83	6.59 ± 4.66	0.21
Age of first cannabis use	16.33 ± 2.78	15.90 ± 2.28	0.71
Age of first regular cannabis use	19.33 ± 2.96	17.20 ± 2.66	0.12
Previous quit attempts	1.67 ± 1.41	2.40 ± 3.89	0.60
AUDIT (*n* = 5,6)	3.60 ± 2.41	4.00 ± 2.37	0.79
FTND	2.56 ± 1.94	3.90 ± 3.28	0.30
Carbon monoxide level (ppm_)	11.56 ± 9.58	18.10 ± 13.30	0.24
*Neurocognitive Tasks edian (range)*			
*Hopkins Verbal Learning Test*			
Delayed recall	8 (2–10)	7 (4–12)	0.71
Percent retention	88.9 (50–133)	87.5 (54.5–116.7)	0.65
Discrimination index	10 (4–12)	11 (6–12)	0.14
*Spatial delayed response*			
5 s delay	19 (8–38)	21 (13–48)	0.81
15 s delay	19 (10–62.3)	22.5 (16.4–68)	0.72
30 s delay	25 (10–80)	25 (19.6–83)	0.49
*Digit Span task*			
Forward	10 (7–16)	10 (4–13)	0.87
Backward	7 (4–10)	6 (3–9)	0.65
*Continuous Performance Test*			
Variability	13.26 (3.18–33.52)	17.57 (4.36–53.05)	0.84
Hit rate	407.47 (345.66–520.63)	372.12 (310.4–431.88)	0.08
Attentional index (d’)	1.52 (−2.48–4.27)	0.56 (−3.46–0.93)	0.08
*Kirby Delay-Discounting Tas*k			
Natural logarithm of Geometric K	−3.67 (−4.19–−3.06)	−2.90 (−6.44–−1.62)	0.36
*Tower of London*			
Total correct	82 (78–132)	88 (80–98)	0.56
Problem solving time	100 (92–104)	95 (76–100)	0.88
Total moves	87 (78–118)	87 (70–96)	0.38
Initiation	99 (92–102)	99 (96–110)	0.88

Participants in the active group received high-frequency (20 Hz) repetitive rTMS applied to bilateral dorsolateral prefrontal cortex 5x/week for 4 weeks. All participants regardless of rTMS treatment condition received weekly behavioral support sessions motivating abstinence from cannabis use (further details in ref. ^19^).

### Neurocognitive tasks

Participants completed the Hopkins Verbal Learning Test (HVLT-R) to assess verbal learning and memory, Spatial Delayed Response task (SDR) to assess delayed visuospatial working memory, the Digit Span (DS) task to assess verbal working memory, the Kirby Delay-Discounting Task (KDDT) to assess delayed discounting and impulsivity, the Continuous Performance Test (CPT) to assess sustained attention, and the Tower of London (TOL) to assess complex planning and problem solving. Specific outcomes assessed and participant scores are reported in Table 1; further details on tasks can be found in ref. ^39^. Note that for the KDDT, Geometric K scores are reported, which describe an individual’s subjective value of delayed rewards, such that larger K values represent steeper discounting of rewards even with small time delays^40^. All participants completed a cognitive training session prior to baseline to reduce practice effects that may emerge between baseline and end of treatment^41^.

### Statistical analysis

Statistical analyses were conducted using Statistical Program for Social Sciences (SPSS®) and R v.4.1.2 (R Core Team, 2021). Demographic and baseline measures for active and sham groups were compared using independent t-tests and chi-square tests. Main treatment effects on clinical and cognitive symptoms are reported elsewhere^19^. Outliers on neurocognitive tasks flagged by boxplot analysis as being three standard deviations above the mean were removed.

To reduce the number of analyses, composite scores were created for HVLT-R outcomes (delayed recall, retention, discrimination index) and SDR outcomes (5 s, 15 s, 30 s delays) by converting raw scores for each outcome to z scores and averaging across all included outcomes. These individual outcomes are conceptually and statistically related and have previously been combined as composites in the literature^42,43^. Remaining outcomes were assessed independently.

Random intercept linear mixed-effects models fit with maximum likelihood using R packages lmer and afex were used to determine interaction effects of time x treatment condition x baseline neuropsychological performance (as a continuous measure) on changes in cannabis GPD and reductions in THC content in urine from baseline to Day 28 (described above). Significance levels were determined with Satterthwaite’s method and estimated marginal means (tukey corrected) in R package emmeans were used to conduct post hoc analyses. Mixed model function in lmer package outputs a t-distributed statistic and degrees of freedom that was then converted to Cohen’s *d* effect size. Results are reported as effect sizes with 95% confidence intervals; significant findings do not contain the null hypothesis value(0). Note that there was a significant difference between groups with respect to age of schizophrenia diagnosis, but this was not related to cannabis or neurocognitive outcomes and thus removed from models for better fit.

With respect to models of change, mediation analyses were conducted using PROCESS^44^ to explore the mediating effect of executive functioning tasks hypothesized to change as a result of non-invasive neuromodulation. Specifically, these were the HVLT-R (percent retention and discrimination index), SDR 30 s delay, CPT (Hit Rate, Variability, Attentiveness), Digit Span (Forward and Backward) and TOL^14,19,45–47^. Given the exploratory nature of this analysis, the dearth of available clinical literature in this area, and the goal of assessing specificity of rTMS treatment effects on neurocognitive performance, composite scores for HVLT-R and SDR were not used. Change scores were calculated by subtracting baseline scores from end of treatment scores; change scores, treatment, and changes in GPD, were then converted to z-scores to obtain completely standardized coefficients. PROCESS uses bootstrapping to output the direct effect, which in this case is the relationship between the rTMS treatment (active) and changes in cannabis use (baseline to day 28), controlling for the specific mediator; the indirect effect (AB), which assesses whether changes in neuropsychological performance (baseline to day 28, pathway A) mediates the relationship between active rTMS and changes in cannabis use (pathway B); and the total effect that sums the direct and indirect effects. Only complete cases were used. The mediation analyses was an exploratory objective for which our study has low power^48^ hence, mediational effects are presented and interpreted in a general way, with focus on estimated effect and their precision, rather than their statistical significance.

## Supplementary information

Supplemental Figure

Supplemental Table

## Data Availability

The data supporting the findings of this study are not publicly available due to ethical restrictions for protecting participants’ confidentiality and privacy but are accessible from the corresponding author on reasonable request with the approval of the Institutional Review Board of CAMH.

## Code availability

No custom code or mathematical algorithm was used for this study.
