# Supplementary material for: Neurocognitive moderation of repetitive transcranial magnetic stimulation (rTMS) effects on cannabis use in schizophrenia: a preliminary analysis
Source: Schizophrenia (Heidelb). 2022 Nov 17;8(1):99. doi: 10.1038/s41537-022-00303-2 (PMC9668838; doi:10.1038/s41537-022-00303-2)
Supplement: Supplementary file 1 — Supplemental Figure [file 41537_2022_303_MOESM1_ESM.pdf]

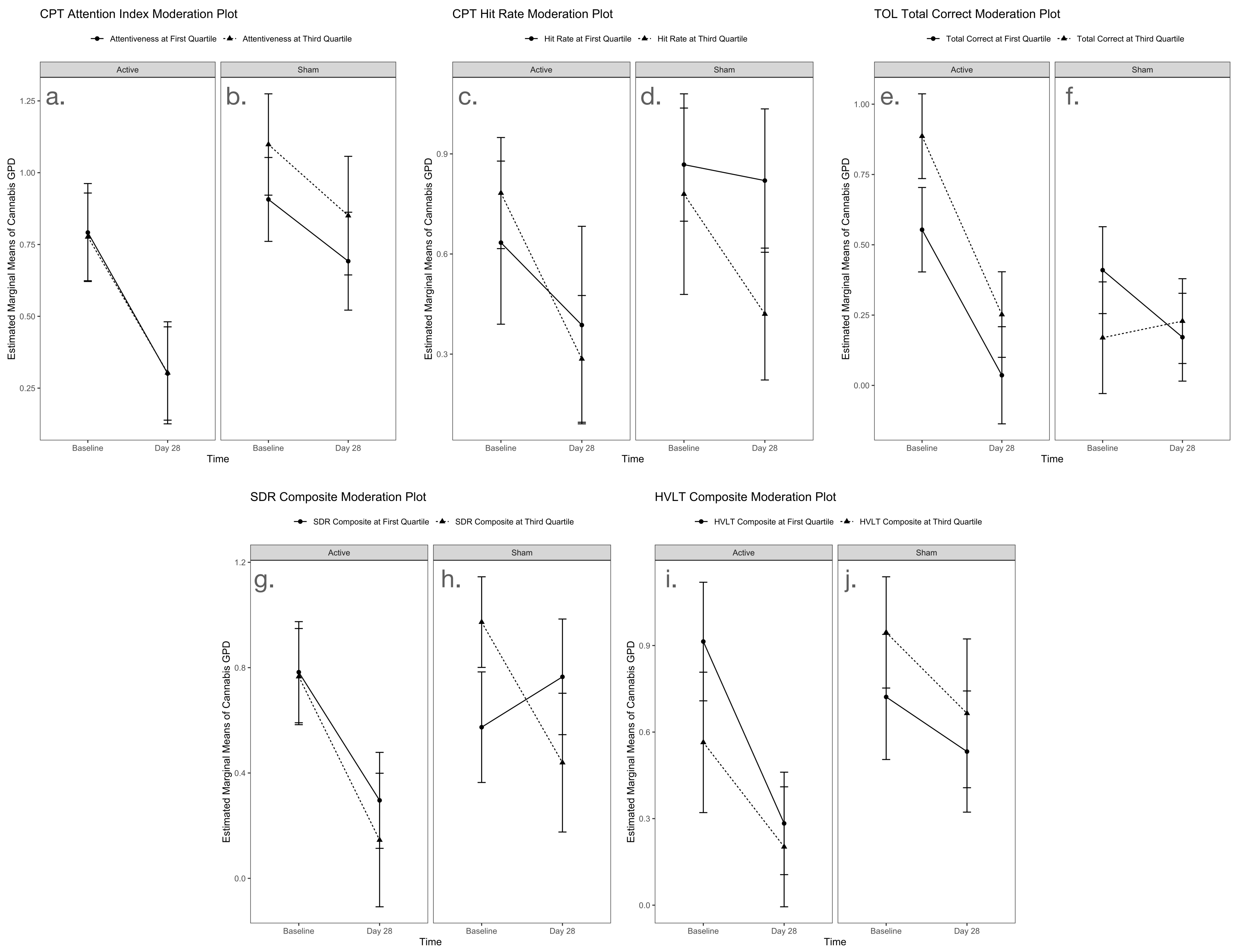

Figure Legend: Figure depicts linear mixed effects models with treatment x time x baseline neurocognitive test performance on changes in cannabis use using estimated marginal means, with standard error estimates. Note that neurocognitive tests were analyzed as continuous variables however the plot depicts cannabis outcomes at high scores (third quartile) and low scores (first quartile) for interpretation. Cannabis use is depicted as estimated marginal means of grams per day.

CPT: Continuous performance test; TOL: Tower of London; SDR: Spatial Delayed Response task; HVLT: Hopkins Verbal Learning Test-Revised
