# Supplementary material for: Neurocognitive moderation of repetitive transcranial magnetic stimulation (rTMS) effects on cannabis use in schizophrenia: a preliminary analysis
Source: Schizophrenia (Heidelb). 2022 Nov 17;8(1):99. doi: 10.1038/s41537-022-00303-2 (PMC9668838; doi:10.1038/s41537-022-00303-2)
Supplement: Supplementary file 2 — Supplemental Table [file 41537_2022_303_MOESM2_ESM.docx]

**Appendix A**

| **Neuropsychological Measure** | **Fixed Effects** | ***Estimate*** | ***SE*** | ***df*** | ***t*** | ***p*** |
| --- | --- | --- | --- | --- | --- | --- |
| **Grams per day** |  |  |  |  |  |  |
| **DS Forward** | intercept | 0.98 | 0.35 | 33.60 | 2.84 | .008** |
|  | Treatment | -0.11 | 0.35 | 33.60 | -0.30 | 0.76 |
|  | Time | 0.23 | 0.25 | 19.72 | 0.93 | .36 |
|  | DS | -0.04 | 0.03 | 34.71 | -1.11 | .27 |
|  | Treatment x Time | 0.24 | 0.25 | 19.72 | 0.99 | .34 |
|  | Treatment x DS | <0.01 | 0.03 | 34.71 | 0.02 | .99 |
|  | Time x DS | < -0.01 | 0.02 | 19.93 | -0.19 | .85 |
|  | Treatment x Time x DS | < -0.01 | 0.02 | 19.93 | -0.75 | .50 |
| **DS Backward** | intercept | 0.71 | 0.27 | 29.74 | 2.62 | .01* |
|  | Treatment | <0.01 | 0.27 | 29.74 | <0.01 | .99 |
|  | Time | 0.32 | 0.19 | 23.00 | 1.66 | .11 |
|  | DS | -0.01 | 0.04 | 30.51 | -0.39 | .70 |
|  | Treatment x Time | 0.04 | 0.19 | 23.00 | 0.22 | .83 |
|  | Treatment x DS | -0.01 | 0.04 | 30.51 | -0.37 | .72 |
|  | Time x DS | -0.02 | 0.03 | 23.59 | -0.68 | .50 |
|  | Treatment x Time x DS | < .01 | 0.03 | 23.59 | 0.10 | .92 |
| **CPT Variability** | intercept | 0.68 | 0.15 | 24.46 | 4.58 | < .001*** |
|  | Treatment | -0.17 | 0.15 | 24.46 | -1.17 | .25 |
|  | Time | 0.15 | 0.10 | 20.09 | 1.51 | .15 |
|  | CPT | <-0.01 | <0.01 | 28.81 | -0.49 | .63 |
|  | Treatment x Time | 0.08 | 0.10 | 20.09 | 0.76 | .46 |
|  | Treatment x CPT | < 0.01 | 0.01 | 28.81 | 0.76 | .45 |
|  | Time x CPT | < 0.01 | < .01 | 24.82 | 0.46 | .65 |
|  | Treatment x Time x CPT | < -0.01 | < .01 | 24.82 | -0.36 | .72 |
| **TOL Initiation Time** | intercept | -0.15 | 1.34 | 16.0 | -0.11 | .91 |
|  | Treatment | 0.53 | 1.34 | 16.0 | 0.39 | .70 |
|  | Time | 0.34 | 0.82 | 10.03 | 0.42 | .68 |
|  | TOL | 0.01 | 0.01 | 16.0 | 0.39 | .70 |
|  | Treatment x Time | -0.48 | 0.82 | 10.03 | -0.58 | .57 |
|  | Treatment x TOL | < -.01 | 0.01 | 16.0 | -0.33 | .75 |
|  | Time x TOL | < -.01 | < .01 | 10.24 | -0.22 | .83 |
|  | Treatment x Time x TOL | < .01 | < .01 | 10.24 | 0.69 | .51 |
| **NarcoCheck (ng/mL)** |  |  |  |  |  |  |
| **DS Backward** | intercept | 356.86 | 131.92 | 32.05 | 2.71 | .01* |
|  | Treatment | -63.44 | 131.92 | 32.05 | -0.48 | .63 |
|  | Time | 102.77 | 81.62 | 22.65 | 1.26 | .22 |
|  | DS | -1.86 | 18.27 | 33.33 | -0.10 | .92 |
|  | Treatment x Time | 43.32 | 81.62 | 22.65 | 0.53 | .60 |
|  | Treatment x DS | 7.36 | 18.27 | 33.33 | 0.40 | .69 |
|  | Time x DS | -6.77 | 11.49 | 23.45 | -0.59 | .56 |
|  | Treatment x Time x DS | -1.80 | 11.49 | 23.45 | -0.16 | .88 |
| **CPT Variability** | intercept | 404.15 | 74.08 | 26.44 | 5.46 | <.001*** |
|  | Treatment | 0.91 | 74.08 | 26.44 | 0.01 | .99 |
|  | Time | 17.87 | 36.23 | 17.78 | 0.49 | .63 |
|  | CPT | -2.49 | 3.25 | 33.16 | 0.77 | .45 |
|  | Treatment x Time | 13.90 | 36.23 | 17.78 | 0.38 | .71 |
|  | Treatment x CPT | 0.08 | 3.25 | 33.16 | 0.02 | .98 |
|  | Time x CPT | 1.46 | 1.95 | 23.73 | 0.75 | .46 |
|  | Treatment x Time x CPT | -0.26 | 1.95 | 23.73 | -0.14 | .89 |
| **TOL Initiation Time** | intercept | 71611 | 870.60 | 15.26 | 0.82 | .42 |
|  | Treatment | 1415.09 | 870.60 | 15.26 | 1.63 | .13 |
|  | Time | 967.07 | 554.20 | 6.30 | 1.75 | .13 |
|  | TOL | -5.02 | 8.70 | 15.34 | -0.58 | .57 |
|  | Treatment x Time | -21.04 | 554.20 | 6.30 | -0.04 | 97 |
|  | Treatment x TOL | -13.75 | 8.70 | 15.34 | -1.58 | .13 |
|  | Time x TOL | -9.79 | 5.61 | 6.54 | -1.75 | .13 |
|  | Treatment x Time x TOL | -0.11 | 5.61 | 6.54 | -0.02 | .99 |

*Note:* Hopkins Verbal Learning Test (HVLT-R), the Spatial Delayed Response (SDR), Continuous Performance Test (CPT), Digit Span (DS), Tower of London (TOL), Kirby Delay-Discounting Task (KDDT)
